# Detection of Antibiotic Resistance in Leprosy Using GenoType LepraeDR, a Novel Ready-To-Use Molecular Test

**DOI:** 10.1371/journal.pntd.0001739

**Published:** 2012-07-31

**Authors:** Emmanuelle Cambau, Aurélie Chauffour-Nevejans, Liana Tejmar-Kolar, Masanori Matsuoka, Vincent Jarlier

**Affiliations:** 1 Université Paris Diderot, EA3964, Paris, France; 2 Centre National de Référence des Mycobactéries et de la Résistance des Mycobactéries aux Antituberculeux, Paris, France; 3 AP-HP, groupe Hospitalier Saint Louis-Lariboisière, Bactériologie, France; 4 Université Pierre et Marie Curie-Paris6, EA1541, ER5, Paris, France; 5 HAIN Lifescience, Nehren, Germany; 6 Leprosy Research Center, National Institute of Infectious Diseases, Higashimurayama-shi, Tokyo, Japan; 7 AP-HP, Groupe Hospitalier Pitié-Salpêtrière, Bactériologie-Hygiène, Paris, France; Fondation Raoul Follereau, France

## Abstract

**Background:**

Although leprosy is efficiently treated by multidrug therapy, resistance to first-line (dapsone, rifampin) and to second-line drugs (fluoroquinolones) was described worldwide. Since *Mycobacterium leprae* is not growing *in vitro*, phenotypic susceptibility testing requires a one year experiment in the mouse model and this is rarely performed. Genetics on antibiotic resistance provide the basis for molecular tests able to detect for antibiotic resistance in leprosy.

**Methodology/Principal Findings:**

A reverse hybridization DNA strip test was developed as the GenoType LepraeDR test. It includes DNA probes for the wild-type sequence of regions of *rpoB*, *gyrA* and *folP* genes and probes for the prevalent mutations involved in acquired resistance to rifampin, fluoroquinolones and dapsone, respectively. The performances of the GenoType LepraeDR test were evaluated by comparing its results on 120 *M. leprae* strains, previously studied for resistance by the reference drug *in vivo* susceptibility method in the mouse footpad and for mutations in the gene regions described above by PCR-sequencing. The results of the test were 100% concordant with those of PCR sequencing and the mouse footpad test for the resistant strains: 16 strains resistant to rifampin, 22 to dapsone and 4 to ofloxacin with mutations (numbering system of the *M. leprae* genome) in *rpoB* (10 S456L, 1 S456F, 1 S456M + L458V, 1 H451Y, 1 G432S + H451D, 1 T433I + D441Y and 1 Q438V), in *folP1* (8 P55L, 3 P55R, 7 T53I, 3 T53A, 1 T53V) and *gyrA* (4 A91V), respectively. Concordance was 98.3% for the susceptible strains, two strains showing a mutation at the codon 447 that in fact was not conferring resistance as shown by the *in vivo* method.

**Conclusions/Significance:**

The GenoType LepraeDR test is a commercially available test that accurately detects for antibiotic resistance in leprosy cases. The test is easy to perform and could be implemented in endemic countries.

## Author Summary

Although leprosy is a curable disease using a combination of antibiotics for one year, the transmission is still active with 230,000 new cases in 2010. Drug resistance has been described and may prevent eradication of the disease. The infectious agent causing leprosy, *Mycobacterium leprae*, is not growing *in vitro* and antibiotic susceptibility testing is possible only in the mouse footpad model that requires a one year experiment. Consequently this testing is rarely done and antibiotic resistance rates in leprosy are unknown. This is the reason why we endeavored to set a new diagnosis test that detects for antibiotic resistance in *M. leprae*. The test is based on the method of a DNA strip test with a multiplex PCR followed by reverse hybridization. It was developed as an easy-to-use test and it will be available in endemic countries, where these kinds of strip tests are already used for detection of drug resistance in tuberculosis. The results of the new test, Genotype LepraeDR, performed on 120 *M. leprae* strains were concordant with those of the standard PCR sequencing and mouse footpad susceptibility testing.

## Introduction

Leprosy, the second communicable disease due to mycobacteria after tuberculosis, is still a preoccupying disease as 230 000 new cases have been reported in 2010 (www.who.int/lep/). This disease remains difficult to diagnose and treat in low- and mid-developed countries, especially in rural areas. Global child rate has remained consistently at around 10% of cases for the last years, showing that transmission is still active [1]. Leprosy can be cured if multidrug therapy (MDT) is properly implemented following WHO recommendations: a 6 month regimen for paucibacillary cases and a 12 month regimen (formerly 24-months) for multibacillary (MB) cases both combining rifampin and dapsone, plus clofazimine for MB cases [2]. The relapse rate ranges between 2% and 5% in leprosy depending of the country, and we learned from tuberculosis that relapse cases are at risk of drug resistance [3]. However, in contrast to what we know for tuberculosis, the prevalence of primary and secondary resistance is unknown for leprosy. Consequently, the risk of resistance cannot be assessed and re-treatment regimen cannot be appropriately design.

*Mycobacterium leprae* is one of the few bacteria that are not growing *in vitro*. It multiplies only in the mouse footpad [4] and in the nine-band armadillo [5]. The in vivo susceptibility testing model, based on footpad inoculation of mice treated with antibiotics, is available in only an handful of highly specialized laboratories and cannot be spread because it requires one year lasting experiment (*M. leprae* doubling time is about 10 to 14 days) and expensive facilities [4], [6]. Resistance to anti-leprosy drugs, such as dapsone, rifampin and fluoroquinolones, has been described since 1967 using this in vivo model [6]. Multi-drug resistance, i.e. resistance to at least two of these drugs, has been described in Africa [7], Asia [8] and South America (unpublished data).

In the late 1990's, thanks to PCR and determination of the *M. leprae* genome [9], molecular methods detecting antibiotic resistance have been set. Rifampin resistance was associated to mutations in the *rpoB* gene encoding the β subunit of RNA polymerase [10], dapsone resistance to mutations in the *folP1* gene encoding the dihydropteroate synthase [11], [12] and fluoroquinolone resistance to mutations in the *gyrA* gene encoding the subunit A of DNA gyrase [7]. Various methods have been described to detect the mutations listed above such as PCR- sequencing, heteroduplexes formation, and DNA array [13], [14], [15], [16], [17], [18]. However, all these methods require specialized laboratories and are not commercially available. No easy-to-use methods are available in the endemic areas.

The DNA strip assay is a methodology widely used for molecular detection of resistance in tuberculosis [19]. The test is based on a classic PCR and reverse hybridization. Because this methodology has been demonstrated to be simple and robust in developing countries, we aimed to develop a new test based on this technology that easily detect for drug resistance in leprosy.

## Materials and Methods

### *M. leprae* strains

Hundred and twelve skin biopsies containing *M. leprae* were studied for the evaluation of the test. They have been sent for leprosy diagnosis to the National Reference Center for mycobacteria (NRC-Myc, Paris, France) from 1989 to 2010 and were all smear-positive for acid fast bacilli (AFB) with a minimum amount of 5×10^4^ AFB/ml. The samples were anonymized and the collection was used under the IRB approval for diagnosis specimens received at Assistance publique Hôpitaux de Paris, Biology laboratories of Pitie-Salpetriere Hospital. The selected biopsies (54% of the collection) were consecutive biopsies for which mouse culture was performed and for which enough quantity of specimen was available for performing the molecular studies. Skin biopsies were prepared as described previously for mouse inoculation and molecular experiments [17], [20].

Eight *M. leprae* strains, which were previously described and propagated in the nude mouse footpad, were taken as reference strains [8], [21].

DNA from several mycobacterial strains other than *M. leprae* were tested for analytical specificity: 3 *M. ulcerans*, 5 *M. marinum*, 5 *M. chelonae*, 1 *M. scrofulaceum*, 1 *M. kansasii*, 1 *M. intermedium*, 1 *M. terrae*, 1 *M. malmoense*, *1 M. fortuitum*. In addition, ten biopsies known to be negative for mycobacteria were also tested for specificity.

### GenoType LepraeDR probe description

The design of the mutated (MUT) and wild type (WT) probes were based on the mutations reported in the literature for the resistant strains: in the rifampin resistance determining region (RRDR) in *rpoB* [10], [17], [22], in the region determining dapsone resistance (DRDR) in *folP1* [11], [12], [20] and in the quinolone resistance determining region (QRDR) in *gyrA* [7], [23]. The probes are listed in Table 1. Wild type probes, one to four according to the gene, were chosen to span the region affected by drug resistance mutations: WT1 to WT4 for *rpoB* (the 430–458 region, numbering system of the *M. leprae* genome TN, GenBank n°NC 002677), WT *folP1* for the 53–55 region and WT *gyrA* for the 89–91 region. Some of the most prevalent mutations in *rpoB* (S456L and H451Y), in *folP1* (P55L) and in *gyrA* (A91V) were included in the strip as specific probes.

**Table 1:** Probes and primers used in the GenoType Leprae DR test for molecular detection of antileprosy resistance.

Antibiotic	Gene	Probe	Targeted codon(s) or mutation*
Rifampin	*rpoB*	WT1	432
		WT2	438–441
		WT3	451
		WT4	456–458
		MUT1	S456L
		MUT2	H451Y
Ofloxacin	*gyrA*	WT	89–91
		MUT	A91V
Dapsone	*folP1*	WT	53–55
		MUT	P55L

na, non applicable.

* numbering system used in the *M. leprae* genome of strain NT (sequence NC 002677 in GenBank).

### GenoType LepraeDR testing

Strips were coated at Hain Lifescience factory (Nehren, Germany) with the different specific oligonucleotides (DNA probes) using the DNA strip technology. Amplification, hybridization and interpretation were performed in a similar procedure as for other GenoType tests [19]. Briefly, 35 µl of 5′-biotinylated primers and nucleotide mix, 5 µl of polymerase buffer, 2 µl of 25 mM MgCl_2_ stock solution, 3 µl of water and 5 µl of total DNA (20 to 100 ng) were mixed with 1 U of Hot Star Taq polymerase (Qiagen) per reaction. The PCR run was comprised of 35 cycles. After denaturation at 95°C for 15 min, ten cycles at 95°C for 30 sec and at 58°C for 2 min were followed by 25 cycles with a first step at 95°C for 25 sec, a second step at 53°C for 40 sec and a third step at 70°C for 40 sec. PCR ended with 8 min at 70°C. Hybridization was performed using the TwinCubator at a temperature of 45°C. The denaturation solution was mixed with 20 µl of the amplified sample and submitted to the usual protocol for hybridization.

In order to assess positive and negative bands, each DNA strip was stuck on an evaluation sheet after the hybridization, and a template was aligned side by side of the respective strip, with at the top the conjugate control band and at the bottom the coloured M marker band. Positive control bands, i.e. that should appear positive to make the test valid, were the conjugate control, the amplification control, the identification control for the *M. leprae* species and amplification controls of the *rpoB*, *folP1* and *gyrA* genes.

Interpretation was as follows for each gene/antibiotic: the strain was predicted to be susceptible when all WT bands were positive and all MUT bands were negative; the strain was predicted to be resistant when at least one MUT band was positive or at least one WT band was negative.

### DNA extraction and reference PCR-sequencing

PCR sequencing was performed routinely and prospectively in the frame of NRC-Myc activities, as individual susceptibility to rifampin (*rpoB*) and dapsone (*folP1*) for all the 112 biopsies whereas ofloxacin susceptibility was tested for 52 biopsies. PCR sequencing was performed specifically in the frame of the present study for the 8 reference strains.

Total DNA was extracted from biopsies containing *M. leprae* following the heat-shock procedure [24]. DNA was subjected to three PCRs, one amplifying the RRDR in *rpoB* gene, one the DRDR in *folP1* and one the QRDR in *gyrA*, as previously described [10], [25]. Typical reaction mixtures (50 µl) contained 1× reaction buffer, 1.5 mM of MgCl_2_, 200 µM of dNTPs, 1 µM of each primer (Proligo France SAS), 1.25 U of *Taq* polymerase (Q-Biogene, Illkirch, France) and 5 µl of DNA extract. PCR-amplified fragments were purified by using Montage™ PCR Centrifugal Filter Devices (Millipore, Molsheim, France) and sequenced by the dideoxy-chain termination method with the ABI PRISM BigDye Terminator v3.1 Cycle Sequencing Kit (Applied Biosystems, Courtaboeuf, France). The oligonucleotide primers used for DNA sequencing were those used for PCR. The nucleotide and deduced amino acid sequences were analyzed with the Seqscape v2.0 software (Applied Biosystems).

### Antibiotic susceptibility testing in the mouse

Animal experiments were performed in accordance with prevailing regulations regarding the care and use of laboratory animals by the European Commission. The experimental protocol was approved by the Departmental Direction of Veterinary Services in Paris, France.

The *M. leprae* strains were subjected to the mouse footpad susceptibility testing that included 10 untreated Swiss mice as a control group, a rifampin-treated group of 8 mice and a dapsone-treated group of 30 mice as described previously [17],[20]. Dapsone susceptibility testing was stopped in 2001 because of new governmental regulation for antibiotic-free animal feeding. An additional group of 8 ofloxacin-treated mice was inoculated, as described in [7], for the biopsies sampled in patients who have been treated by fluoroquinolones.

### Evaluation of the diagnosis performances

The results of the GenoType LepraeDR test were compared to those of the PCR sequencing method for all the 120 *M. leprae* strains (60 in the case of ofloxacin and *gyrA*).

The results of the GenoType LepraeDR test were also compared to the results of the mouse footpad model for *M. leprae* strains that yielded interpretable susceptibility results, i.e. 84 strains tested in vivo for rifampin susceptiblity, and among them 56 for dapsone susceptibility and 5 for ofloxacin susceptibility.

## Results

### Performances of GenoType LepraeDR for detection of *M. leprae*

The DNA strip tests were validated with regard to the *M. leprae* identification band, which was positive with an intensity equal or higher than that obtained with the universal positive control, demonstrating the presence of *M. leprae* DNA. Thus, the overall sensitivity of GenoType LepraeDR for detecting *M. leprae* was 100%.

Analytical specificity tested with either DNA from another mycobacterial species (n = 19) or negative skin biopsies (n = 10) was 100% since no positive signal was obtained for the *M. leprae* identification band. However, hybridization was observed for DNA from *M. intermedium* and *M. malmoense* with two of the wild type *rpoB* bands, due to a high identity between the *rpoB* genes of these mycobacterial species.

### Performances of GenoType LepraeDR for detecting mutations in the genes involved in antileprosy drug resistance

**Figure 1:**
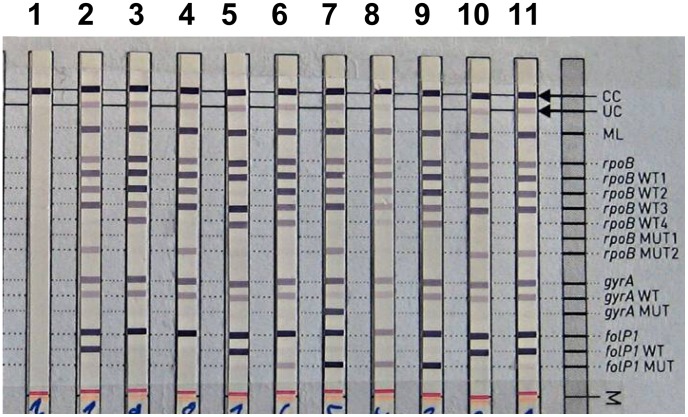
Mutations conferring resistance in *Mycobacterium leprae* are detected by the GenoType LepraeDR DNA strip test. Lane 1 is a negative control (only the CC band). Lanes 2 to 11 showed various profiles for resistant strains: lane 2, *rpoB* mutation S456L with wild type *gyrA* and *folP1* alleles; lane 3, wild type *rpoB* and *gyrA* alleles with a *folP1* mutation to be defined; lane 4, *rpoB* mutation S456L with a wild type *gyrA* allele but a mutation in *folP1*; lane 5, *rpoB* mutation (Q438V) with wild type *gyrA* and *folP1* alleles; lane 6, wild type *rpoB* and *gyrA* alleles with a P55L mutation in *folP1*; lane 7, *rpoB* mutation S456L with a A91V *gyrA* mutation and a P55L mutation in *folP*; lane 8 and lane 9, wild type *rpoB* and *gyrA* alleles with a P55L mutation in *folP1* ; lane 10 and lane 11, *rpoB* mutation S456L with wild type *gyrA* and *folP1* alleles. The numbering system used is that of the *Mycobacterium leprae* genome strain NT (n°NC 002677).

**Figure 2:**
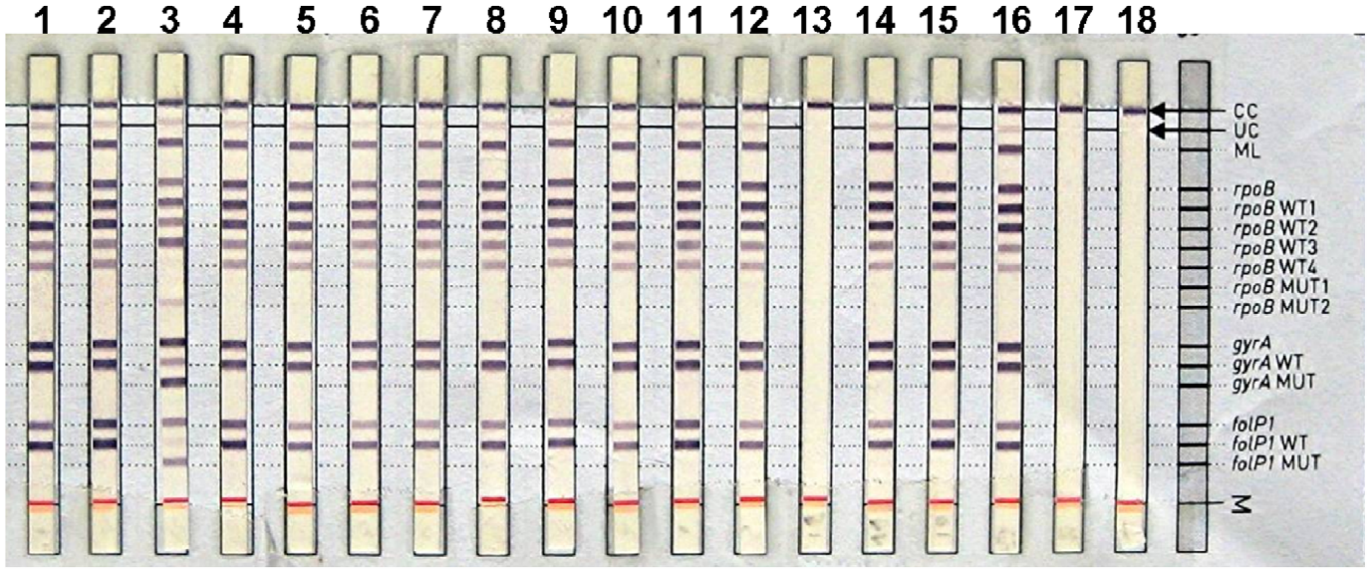
*Mycobacterium leprae* susceptible strains showed a wild type profile in the GenoType LepraeDR test. Lane 1 to 16 (except lane 8) showed wild type profiles for susceptible *M. leprae* strains. Lane 8 showed a multiresistant profile with mutations in *rpoB*, *gyrA* and *folP1* genes. Lanes 17 and 18 showed result of negative controls.

The mutations found in the *M. leprae* strains by PCR-sequencing are listed in the Table 2. Representative results of the DNA strip tests are shown in Figure 1 for resistant strains and in the Figure 2 for susceptible strains.

**Table 2:** List of mutations present in the *M. leprae* resistant strains.

Mutations in the region determining resistance in the following genes (N strains)		
*rpoB*	*folP1*	*gyrA*
S456L (10)	P55L (8)	A91V (4)
S456F (1)	P55R (3)	
S456M + L458V (1)	T53I (7)	
H451Y (1)	T53A (3)	
G432S + H451D (1)	T53V (1)	
T433I + D441Y (1)		
Q438V (1)		

The results of the DNA strip test were concordant with those of PCR sequencing for all the 16 *rpoB* mutations conferring rifampin resistance (Table 3). We observed a positive signal at probes rpoBMUT2 for the 10 strains harboring the mutation S456L and at rpoBMUT1, for the strain harboring the H451Y mutation, since these mutations are present onto the strip as a mutated probe. As expected for these strains, no signals were observed for the wild type probes rpoBWT4 and rpoBWT3, respectively. For the others mutations, the test detected the *rpoB* mutation through the lack of hybridization with the wild type probes that include the mutated codon (Table 1), e.g. with rpoBWT4 for the two strains harboring the mutation S456M or S456F, with rpoBWT2 for the strain with the mutation Q438V, rpoBWT1 and rpoBWT3 for the strain harboring the two mutations G432S + H451D and rpoBWT1 and rpoBWT2 for the strain harboring the two mutations T433I + D441Y. For two strains carrying a mutation at the codon 447, they were not detected by the DNA strip test since no probe spanning this codon is included in the strip because this mutation was not known to confer resistance. The first of these strains showed a silent mutation and the second showed a mutation leading to the substitution S447C. Although the latter strain appeared susceptible to rifampin in the routine mouse footpad testing, we repeated this test using decreasing dosages of rifampin in order to be sure that the S447C mutation does not confer resistance in *M. leprae* as a similar mutation does in *M. tuberculosis* [26], even at a low level. For this purpose, three groups of mice (10 mice per group) were treated by 10 mg/kg (normal dosage), 5 mg/kg or 2.5 mg/kg rifampin. Growth was not observed in any of these groups but occurred in the control untreated group, demonstrating that the strain was really susceptible to rifampin and that the S447C mutation was not conferring resistance. Moreover, the patient, who was an immigrant from Senegal, was cured after being treated by the standard MDT, i.e. the combination of rifampin, dapsone and clofazimine. For the other 102 other strains, no mutations were detected by the RRDR sequencing in *rpoB* and the DNA strip test.

**Table 3:** Concordance of results for the DNA strip test (GenoType LepraeDR) and the susceptibility phenotypic and genotypic pattern of antibiotic resistance for the *M. leprae* strains studied.

*M. leprae* strains	N diagnosis tests with interpretable results			Concordance GenoType LepraeDR N strains (%)	
	In vivo susceptibility testing*	PCR sequencing	DNA strip test	versus in vivo Susceptibility testing	versus PCR sequencing
Total tested for at least one antibiotic	84	120	120	84 (100%)	120 (98%)
Rifampin resistant	13	16	16	13 (100%)	16 (100%)
Rifampin susceptible	71	104	104	71 (100%)	102** (97%)
Dapsone resistant	8	22	22	8 (100%)	22 (100%)
Dapsone susceptible	48	98	98	48 (100%)	98 (100%)
Ofloxacin resistant	1	4	4	1	4
Ofloxacin susceptible	4	56	56	4	56 (100%)

* For strains growing in vivo and yielding interpretable susceptibility results. Tests were stopped for dapsone due to new regulation for antibiotic animal feeding. Tests for ofloxacin were restricted to patient with previous treatment by fluoroquinolones.

** including two strains with a mutation at codon 447: Ser447Cys for one strain and a silent mutation for the second strain (see text for details).

Concordance was observed between the DRDR sequence in *folP1* and the DNA strip test: 22 strains with a *folP1* mutation involved in dapsone resistance and 98 strains with a wild-type *folP1* sequence (Table 3). Hybridization was observed with the *folP*1 MUT probe for the 8 strains with the *folP1* P55L mutation. For the 14 strains harboring other mutations at codon 55 (P55R) or at codon 53 (T53I, T53A, T53V), there was no signal with the wild type probe, showing that there was a mutation.

Finally, we observed a concordance between the QRDR sequence in *gyrA* and the DNA strip test results: 56 strains with a wild type sequence showed a *gyrA* WT band and the four strains with the mutation A91V showed the *gyrA* MUT band (Table 3).

### Concordance between susceptibility phenotype and genotype determined by the DNA strip test

Concordance was observed between the phenotypic susceptibility results assessed by the mouse footpad model and the genotype detected by the GenoType LepraeDR test. Results are detailed in Table 3 with regard to the antibiotic tested.

Concordance between rifampin phenotypic susceptibility in vivo and the results of GenoType LepraeDR was obtained for all the 84 strains tested. Thirteen rifampin-resistant strains showed either the rpoBMUT1 band (S456L) for 9 strains, or the absence of at least one rpoB WT band for the remaining 4 strains, which indicated a mutation in the RRDR. The exact nature of the *rpoB* mutation was further identified by PCR-sequencing. All the 71 susceptible strains were founded susceptible by the DNA strip test since all the rpoB WT bands were positive and all of the MUT bands were negative.

Concordance between dapsone phenotypic susceptibility and detection of *folP1* mutation by the DNA strip test was obtained for the 48 susceptible and the 8 resistant strains. For all the resistant strains, the folP WT band was negative, indicating a mutation in the DRDR. The folP MUT band was positive for two of these strains, indicating a mutation P55L. In the 6 remaining strains, the exact nature of the *folP* mutation was identified by PCR-sequencing. For the 48 dapsone-susceptible strains, the folP1 WT band was positive and the MUT band was negative

Finally, results of ofloxacin phenotypic susceptibility were concordant with the results of *gyrA* obtained by the DNA strip test for the five strains tested in the mouse footpad: one was resistant and showed a positive gyrA MUT band (mutation A91V) with a negative WT band, and the four susceptible strains showed a positive gyrA WT band and a negative MUT band.

## Discussion

Leprosy, after centuries of endemicity when the disease lasted the whole patient life due to a lack of efficient treatment, became a curable disease by combining rifampin and dapsone into a multidrug therapy regimen [2]. Consequently, a dramatic decrease in the prevalent active cases occurred during the two last decades. However, the incidence rate did not decrease showing that leprosy is still an actively transmitted disease [1]. Acquired resistance has been observed for each of the antileprosy drugs following their successive introduction as antileprosy agent [27], [28]. Multidrug resistant strains resulting from the accumulation of distinct resistant traits have been described in several endemic regions [7], [22]. Proportions up to 80% of secondary resistance (patients previously treated) and 40% of primary resistance (patients never treated) to dapsone and up to 40% secondary resistance to rifampin, have been reported through local and limited studies [28], [29], [30]. Since *M. leprae* is not growing in vitro, it is not possible to measure resistance rates at large scale in endemic countries. Even in highly specialized leprosy centers where the animal model has been set up, it is nowadays very difficult to sustain animal facilities because of ethic rules and safety measures. Molecular detection of resistance to antileprosy drugs has been introduced since genetic bases of resistance were deciphered by expert scientific labs in France, US and Japan Cambau 1997 [10], [11], [12], [31]. We previously showed that mutations in the target genes in clinical *M. leprae* strains were associated with acquired resistance demonstrated by in vivo drug susceptibility testing: in *rpoB* for rifampin resistance, in *folP1* for high and medium level dapsone resistance, and in *gyrA* for ofloxacin resistance [7], [17], [20]. These studies demonstrated concordance between genotypic and in vivo phenotypic results. Therefore, in-house molecular detection is being used for individual diagnosis of leprosy cases in countries where PCR sequencing is affordable [15], [31], [32], [33], [34], [35], [36].

Following years of using various in house molecular methods to rapidly detect for drug resistance in *M. tuberculosis*, particularly to detect for multi-drug resistant cases, i.e. cases resistant to isoniazid and rifampin that cannot be cured by the standard regimen, standardized and commercially available kits, such as the line probe assays, InnoLiPA Rif.Tb and GenoType MTBDR, and more recently GeneXpert RifTB, have been introduced and are recommended in low-income but highly epidemic countries (www.who.int/tb/strategy/en/).

WHO launched in 2008 a programme of surveillance of drug resistance in leprosy using molecular methods relying on a handful of national and supranational reference laboratories. First results obtained for cases reported in 2008, 2009 and 2010, showed that rifampin, dapsone and fluoroquinolone resistance were described but the resistance rates varied from 0 to 10% [37]. This needs confirmation at a larger scale and for an extended time. However this showed that the rates of resistance to antileprosy drugs can be measured by using molecular methods.

The DNA strip technology has been developed as GenoType kits and applied to the molecular detection of antibiotic resistance in various infections such as tuberculosis and *Helicobacter pylori* diseases [19], [38]. This approach has been shown to be easy to use, requiring only a classic thermocycler and a hybridization chamber at a constant temperature of 45°C. This is the reason we choose to develop a standardized test based on the DNA strip technology able to detect for molecular detection of resistance in leprosy.

The new test, GenoType LepraeDR, was evaluated by systematically testing 120 *M. leprae* strains studied for genotypic and phenotypic characters of resistance [17], [20], [22]. The results yielded by the test were shown to be 100% concordant with those of the in vivo susceptibility testing whereas the results of PCR sequencing was 98.3% for rifampin, 100% for dapsone, and 100% for fluoroquinolones. Moreover, the two *rpoB* mutations not detected by the test, located at the codon 447, a codon not included in the test, were in fact not conferring rifampin resistance.

We focused deliberately the present evaluation on AFB-positive specimen from multibacillary leprosy cases for two reasons: (i) first the AFB positivity represents a major clue in leprosy diagnosis that allows concentrating subsequent tests on mot probable cases, an important point in low income countries and (ii) second, the risk of developing acquired resistance by selection of resistant mutants are highest in multibacillary cases. We did not evaluate the performances of the test on either AFB-negative specimen nor on specimen other than skin biopsies (e.g. nasal wabs). The specificity of the test with regard to other mycobacterial species involved in skin infections was assessed for Buruli ulcer and infections due to *M. marinum*, *M. chelonae*, *M. abscessus*, *M. fortuitum*, *M. terrae* and other less common mycobacteria. Because of the high identity of the *rpoB* gene between some mycobacterial species, the results of resistance mutation in *rpoB*, *gyrA and folP* genes by the test can be interpreted only when the test identifies the species as *M. leprae* (positive ML band).

Various other methods have been described to detect mutations in *rpoB*, *gyrA* and *folP* such as PCR sequencing, heteroduplexes, and DNA array [13], [14], [15], [16], [18]. There were mostly used in large laboratories affiliated to Universities of high income countries and collecting strains from endemic countries [34], [39]. Since the reverse hybridization technology is already used in several countries endemic for tuberculosis, the same technology could be also used for the diagnosis of resistance in leprosy in countries where leprosy is still a preoccupying disease, with two objectives: (i) diagnosing resistance at the individual level and (ii) assessing rates of secondary and primary resistance in collaboration with health authorities [1], [37]. Although leprosy is now diagnosed in the field using clinical findings only and no laboratory support is available, such a test can be used complementary to the clinical diagnosis of multibacillary leprosy for (i) relapse cases, especially those who have not been treated by MDT, i.e. before 1982, and (ii) survey of resistance in new cases in defined areas or periods for epidemiological surveillance on the behalf of leprosy public health programmes. Therefore the specimen can be send to a regional lab, especially one used to similar molecular test detecting resistance in tuberculosis. In addition, clinical microbiology laboratories in high income countries, which have usually moderate expertise in leprosy diagnosis and resistance detection, would appreciate the robustness of the test, and such a test can help in diagnosing cases from immigrants or national intertropical territories [40], [41]. Using this technology routinely at the French National Reference Center for mycobacteria during the last two years, we diagnosed 35 cases of leprosy in patients living in France and detected 4 cases with dapsone resistant strains (*folP1* mutations as P55L in 3 strains and T53A in one strain) and 1 case with an ofloxacin resistant strain (*gyrA* A91V mutation) (data not shown). These results, obtained independently of the present evaluation, support the practical interest of this technology.
